# Primary mediastinal liposarcoma - computed tomography and pathological findings: a case report

**DOI:** 10.4076/1757-1626-2-8703

**Published:** 2009-08-07

**Authors:** Fabiana Barroso Thomaz, Edson Marchiori, Anderson Nassar Guimarães, Isabela Fernandes de Magalhães, Fabio Vargas Magalhães, Letícia Pereira Gonçalves, Romeu Cortes Domingues

**Affiliations:** 1 CDPI- Clínica de Diagnóstico por Imagem, Centro Médico Barrashopping, Avenida das Américas, 4666, grupos 302A, 303, 307, 325, 326. CEP 22631-004Barra da Tijuca. Rio de JaneiroBrazil; 2 Department of Radiology, Faculty of Medicine, Federal University of Rio de Janeiro. Rua Professor Rodolpho Paulo Rocco, 255Cidade Universitária. CEP 21941-913. Rio de JaneiroBrazil

## Abstract

Liposarcomas are the most common soft tissue sarcoma of adults, and primary mediastinal liposarcomas are rare. We present a case of a 50-year-old man with primary mediastinal liposarcoma without any invasion into the surrounding structures, such as the esophagus, trachea, or left atrium of the heart. Following surgical removal of the liposarcoma, the patient has had no recurrence after one year. Surgical removal is the treatment of choice for a mediastinal liposarcoma; however, careful long-term follow-up is necessary because the recurrence rate is very high.

## Introduction

Liposarcomas are the most common soft tissue sarcoma of adults, usually arising in the extremities or the retroperitoneum. Primary mediastinal liposarcomas are rare, representing less than 1% of all mediastinal tumors [1]; because of this rarity, documentation in the literature is limited. Here we present a case of primary mediastinal liposarcoma in a 50-year-old man that was successfully managed by complete surgical excision.

## Case presentation

**Figure 1.**
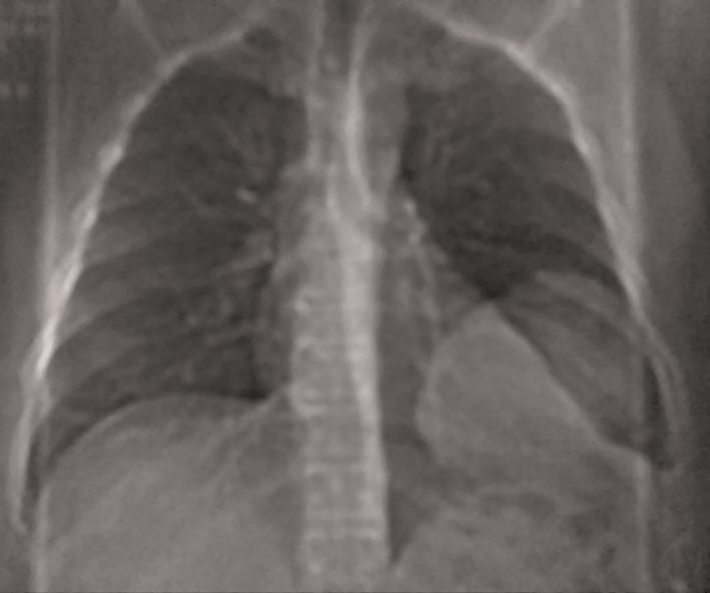
Posteroanterior chest radiograph demonstrates a large, smooth, well-defined mass in the left lower hemithorax.

**Figure 2.**
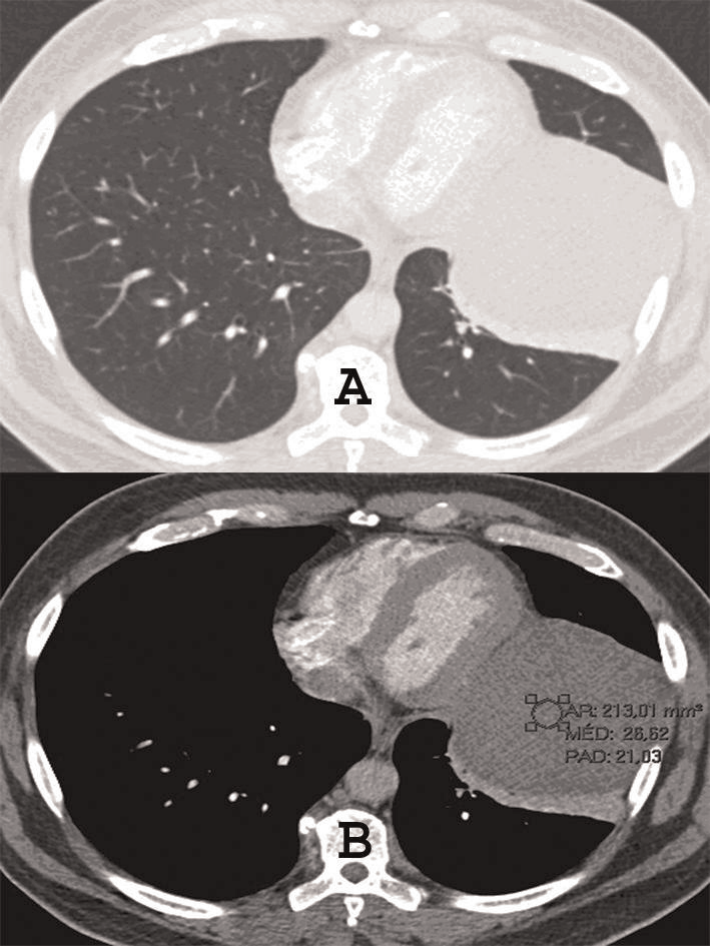
CT with **(A)** mediastinal and **(B)** lung windows shows predominantly a low attenuation mass (+27 UH) without enhancement in the left lower hemithorax.

**Figure 3.**
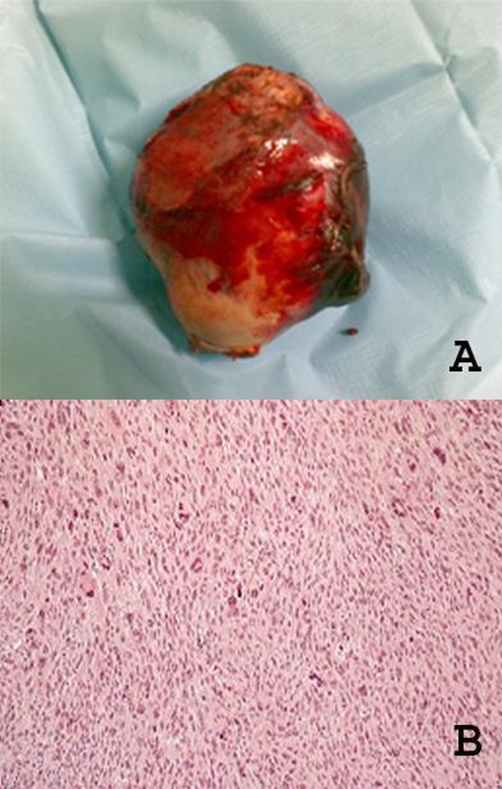
**(A)** Gross appearance of a pleomorphic liposarcoma arising in the mediastinum. **(B)** A histologic section of the resected tumor shows the appearance of pleomorphic liposarcoma (HE ×100).

A 50-year-old Caucasian Brazilian man with left-side chest pain was found to have a mass in the left lower hemithorax in a chest x-ray, and he was referred to our clinic. Physical examination showed dullness on percussion and bilateral decreased breath sounds. Laboratory data, respiratory function tests, and arterial blood gas analyses were within normal limits. The chest x-ray demonstrated a well defined mass in the left lower hemithorax (Figure 1). Chest computed tomography (CT) demonstrated a large, smooth, well-defined mass with soft tissue density (+27 UH) in the basal region of the left hemithorax (Figure 2). Cranial and abdominal CT to detect distant metastasis showed no abnormal findings. Complete surgical excision was attempted. The tumor was found to contain multiple individually encapsulated locules without any invasion into surrounding structures such as the esophagus, trachea, or left atrium of the heart. The tumor measured 12.5 × 11.5 × 7.0 cm. Histological findings showed pleomorphic liposarcoma (Figure 3). The patient has had no recurrence for one year following this operation.

## Discussion

Liposarcomas most often present in either the deep soft tissue of the limbs or the retroperitoneum, although the overall anatomic distribution is widespread. Although liposarcoma is among the most common soft tissue sarcoma overall, intrathoracic liposarcoma is very rare, representing less than 3% of all liposarcomas [2]. Nine percent of the primary sarcomas in the mediastinum are liposarcomas [3].

In the study of mediastinal liposarcoma by Hahn [4], the average age of patients at the time of diagnosis was 51 years. This is essentially identical to the mean age of patients with liposarcomas of the retroperitoneum or extremities [5]. Although liposarcoma is primarily a disease of adults, previous studies have reported that a significant percentage of mediastinal liposarcomas occur in children and young adults. Klimstra et al [6] found that the average age of patients with anterior mediastinal liposarcomas was 43 years, with 30% between 20 and 35 years of age.

The presenting signs and symptoms are related to tumor size and the direct invasion of contiguous structures. Dyspnea, tachypnea, and wheezing are the most common symptoms, followed by chest pain [7]. Asymptomatic cases discovered by radiological imaging also have been reported [8]. Pathologically, liposarcomas are categorized into five groups: well-differentiated, myxoid, round cell, dedifferentiated, and pleomorphic. The clinical behavior of a given liposarcoma correlates with its histological pattern. The well-differentiated forms are of low-grade malignancy and rarely metastasize. The poorly differentiated ones are often highly aggressive in behavior and they tend to recur and produce metastases in a higher percentage of reported cases. The survival in patients with dedifferentiated or pleomorphic liposarcomas was significantly shorter than in patients with myxoid or well-differentiated liposarcomas [9].

The appearance of mediastinal liposarcomas in CT varies from a predominantly fat-containing mass to a solid mass [10]. Density is related to tumor heterogeneity and the extent of necrosis and the soft tissue component in the liposarcoma [11]. T1-weighted magnetic resonance images show the fat tissue with high signal intensity, whereas the signal intensity diminishes in T2-weighted images. Pleomorphic types showed a markedly heterogeneous internal structure.

Total resection of mediastinal liposarcomas is desirable; however, most tumors arise in surgically inaccessible locations. Thus radiotherapy and chemotherapy may be added as adjuncts to surgical excision, although liposarcomas seem to have low sensitivity to these treatments [11]. Recurrence is common in deep-seated liposarcomas; in most cases, it becomes apparent within the first 6 months, but it may be delayed for 5 to 10 years following the initial excision. In conclusion, although liposarcoma is one of the most common soft-tissue sarcomas in adults, mediastinal liposarcoma is rare. Because the recurrence rate after treatment of mediastinal liposarcoma is very high, careful long-term follow-up is necessary.

## Abbreviation

CT: computed tomography
